# Parasites in Mexican patients with irritable bowel syndrome: a case-control study

**DOI:** 10.1186/1756-3305-3-96

**Published:** 2010-10-13

**Authors:** Maria Elena Ramirez-Miranda, Rosaura Hernandez-Castellanos, Eduardo Lopez-Escamilla, David Moncada, Alfredo Rodriguez-Magallan, Carlos Pagaza-Melero, Alberto Gonzalez-Angulo, Ana Flisser, Simon Kawa-Karasik, Pablo Maravilla

**Affiliations:** 1 Dr. Manuel Gea Gonzalez General Hospital, Mexico, DF 14080, Mexico; 2 Hospital Juarez de Mexico, Mexico, DF 07760, Mexico; 3 Departamento de Microbiologia y Parasitologia, Facultad de Medicina, Universidad Nacional Autonoma de Mexico, Mexico DF 04510, Mexico

## Abstract

One hundred and fifteen patients with symptoms suggestive of irritable bowel syndrome (IBS) according to Rome III criteria and 209 patients with gastrointestinal symptoms different from IBS (control) were identified through medical records from the Gastroenterology Clinic of the "Dr. Manuel Gea Gonzalez General Hospital" from January 2008 to March 2010. No statistical differences in IBS data as compared with control groups were observed except in bloating, that was more frequent in the IBS group (*P *= 0.043). Although the pathogenicity of specific intestinal protozoa could not be demonstrated due to lack of association with the development of gastrointestinal symptoms, *Blastocystis spp*, in the IBS group, exhibited a trend of association to diarrhoea (odds ratio = 2.73, 95% confidence interval = 0.84-8.80, *P *= 0.053), while having any parasite and diarrhoea was significant (odds ratio = 3.38, 95% confidence interval = 1.33-8.57, *P *= 0.008). The association between *Blastocystis *and diarrhoea in IBS patients although not conclusive is an interesting finding; nonetheless more extensive case-controlled studies are required to clearly define the role of some "non-pathogenic" parasites in intestinal disease and IBS.

## Findings

In recent times, some common "non-pathogenic" parasites such as *Blastocystis hominis *and *Diantamoeba fragilis *have been associated with abdominal pain, bloating, and alteration of bowel habits resembling irritable bowel syndrome (IBS) [1,2]. IBS is a functional bowel disorder in which abdominal pain or discomfort is associated with defecation disorders [3]. IBS is a frequent disease in clinical practice, representing 12% of all diagnoses obtained in general medical visits and 25% to 50% of all gastroenterology consultations [3,4]. The objective of the present study was to determine the role of intestinal protozoa among IBS patients and to analyze the clinical course of the disease.

Individuals who had attended the gastroenterology clinic of the "Dr. Manuel Gea Gonzalez General Hospital" between January 2008 and March 2010 were identified from the database of medical records. The study was reviewed and approved by the Ethical and Research Committee of the hospital. Cases were defined as patients with symptoms of IBS according to the Rome III criteria. Such symptoms would consist of recurrent abdominal pain or uncomfortable sensation at least 3 days per month in the last 3 months associated with 2 or more of the following: i) improvement with defecation, ii) onset associated with a change in frequency of stool release, iii) onset associated with a change in form or appearance of stools [3]. The control group consisted of individuals without diagnosis of IBS but with other physiological alterations and gastrointestinal symptoms i.e., polypus, diabetes, haemorrhoids, ulcerative colitis, etc. Coprological studies were performed using Faust's technique with three faecal samples [5,6]. Descriptive statistics were expressed as mean and standard deviation (SD). Variables were analyzed by χ^2 ^tests for categorical variables; odds ratio and confidence interval were also obtained. Data analysis was performed with SSPS Version 15.0 for Windows (SPSS Institute, Chicago, IL).

A total of 253 female and 112 male patient records were analyzed during the two-year study period. The mean age was 52 (SD = 15) years; all patients resided in urban areas. Table 1 shows the results and a statistical analysis of clinical signs and symptoms and parasites in the IBS vs. the control group. No statistical differences in both groups were observed except in bloating, that was more frequent in IBS patients (*P *= 0.043). In both groups, abdominal pain and constipation were in general the predominant symptoms (84% and 62% respectively) and *Blastocystis, Endolimax nana and Entamoeba histolytica/dispar *were the most frequent parasites (21%, 13%, and 7% respectively). Group analysis showed a significant association between having any parasite and diarrhoea in IBS patients, while *Blastocystis *exhibited a trend of association with diarrhoea in the same group (Table 2). In all the parasitized patients, stool was examined before treatment with the drug nitazoxanide according to Diaz *et al. *[7].

**Table 1:** Demographic, clinical characteristics and parasites in IBS patients and in the control group

Characteristic	Frequency		
	IBS group	Control group	*P *value
Age at diagnosis, years	51 ± 15	53 ± 15	0.482
Female/male, n	91/24	162/47	0.736
Clinical symptoms, %			
Abdominal pain	81.7	85.6	0.355
Bloating	84.3	74.6	0.043
Constipation	65.2	60.3	0.381
Diarrhoea	26.1	28.2	0.679
Flatulence	12.2	10.0	0.555
Diarrhoea-Constipation	8.7	5.3	0.229
Parasites found, %			
Any parasite	22.6	19.6	0.524
*Blastocystis spp*	15.7	12.0	0.348
*Endolimax nana*	8.7	6.7	0.511
*Entamoeba histolytica/dispar*	2.6	2.9	0.890
*Giardia lamblia*	1.7	1.4	0.831
Co-infection with 2 parasites	6.0	3.4	0.246

**Table 2:** Association between having any protozoan parasite or *Blastocystis *and diarrhoea

Group (n)	Variable	OR*	95%IC‡	**χ**^**2**^	*P *value
IBS group (115)	Any parasite/Diarrhoea	3.38	1.33-8.57	7.02	0.008
	*Blastocystis*/Diarrhoea	2.73	0.84-8.80	3.74	0.053
Control group (209)	Any parasite/Diarrhoea	0.783	0.36-1.72	0.37	0.542
	*Blastocystis*/Diarrhoea	0.781	0.29-2.06	0.25	0.617
All participants (324)	Any parasite/Diarrhoea	1.39	0.77-2.48	1.22	0.269
	*Blastocystis*/Diarrhoea	1.33	0.62-2.8	0.64	0.420

*OR, Odds ratio; ‡95%IC, 95% Confidence interval.

There are no previous clear reports about association between IBS and parasites in Latin American patients. Our results show that the overall prevalence of intestinal protozoa in Mexican IBS patients was 23% (*Blastocystis spp *in 16%, *E. nana *in 9% and *E. histolytica/dispar *in 3%), these findings suggest an association between IBS and *Blastocystis*. A prior study by Yamamoto-Furusho and Torijano-Carrera [8] explored the prevalence of intestinal protozoa in Mexican patients from urban areas with ulcerative colitis and found a prevalence of 24% for intestinal protozoa (*Blastocystis spp *10%, *E. nana *9% and *E. histolytica *5%); in the present study and in Yamamoto-Furusho's one, *Blastocystis *was the most frequent parasite, in spite of the fact that in both studies different diagnostic techniques (trichrome-staining and concentration by flotation, respectively) were used. Yakoob *et al. *[9] demonstrated that faecal carriage of *B. hominis *occurred more frequently in Pakistani patients with IBS (46%) than the control group (7%). In contrast, Tungtrongchitr *et al. *[10] found no association between faecal carriage of *Blastocystis *and IBS in Thailand participants.

According to some researchers [5,11], *Blastocystis *is currently the main dominant parasite found in human stool samples; it may have displaced other protozoa due the reluctance of physicians to treat the infection, as symptoms are self-limiting, and its pathogenicity has been considered as controversial. An additional factor leading to *Blastocystis*' dominance may be that this microorganism has developed resistance to antiparasitic drugs which treat pathogenic protozoa. Following conventional treatment, intestinal empty niches may be easily colonized by *Blastocystis spp. *Finally, similar to other intestinal protozoa, the presence of deficient hygienic habits and lack of health education programs increase the prevalence of this parasite.

Studies into the clinical significance of *Blastocystis *may be influenced by the sensitivity of detection methods. Conventionally, it is detected using standardized clinical coprological methods, which are also used to detect other enteric parasites [12]. Faecal concentration, direct smears and staining have been used by several authors to detect the presence of *Blastocystis*, although those methods have been shown to have a low sensitivity [11,12], Recently, amplification of specific DNA directly from faeces provides a significantly higher sensitivity than concentration techniques and is 100% specific [12,13], although this molecular technique is not commonly available in developing countries.

The pathophysiology of IBS remains elusive because no mechanism is unique to, or characteristic of IBS. There are no firm recommendations about the extent and type of testing required to exclude other pathologies in this disease; investigation of stool for ova, cysts and parasites is generally recommended when diarrhoea is the major manifestation of IBS, but the role that protozoan parasites may play in this complex disease has not been fully investigated [1,3,14]. Furthermore, prior studies had found that IBS patients exhibit certain genetic polymorphisms related to IL-10, TNF-alpha, IL-4, and serotonin [15,16]; thus, we can hypothesize that when patients with certain cytokine gene polymorphisms are infected with *Blastocystis*, they are more likely to develop diarrhoea. Although our results showed a trend of association between this parasite and diarrhoea in IBS patients, more extensive case-control studies that include parasitological molecular diagnostic techniques and genetic profiles of pro-inflammatory cytokines are required to clearly define the potential role of "non-pathogenic" intestinal parasites.

## Abbreviations

IBS: Irritable Bowel Syndrome; CPS: Coproparasitoscopic studies; SD: Standard deviation.

## Competing interests

The authors declare that they have no competing interests.

## Authors' contributions

MERM, RHC and AGA attended patients in the gastroenterology clinic of the "Dr. Manuel Gea Gonzalez General Hospital" and collected data. ELE and DM performed CPS. ARM, CPM, AF and SKK participated in the discussion. MERM and PM formulated the idea and PM performed the statistical analysis. All authors contributed in writing the manuscript.
